# The Role of English as a Foreign Language Teachers’ Technological Pedagogical Content Knowledge on English as a Foreign Language Students’ Achievement

**DOI:** 10.3389/fpsyg.2022.946081

**Published:** 2022-06-28

**Authors:** Guoxiang Duan, Linlin Jia, Hui Chen

**Affiliations:** ^1^School of Foreign Languages, Zhongkai University of Agriculture and Engineering, Guangzhou, China; ^2^Department of Chinese Language and Literature, Xiamen University, Xiamen, China

**Keywords:** EFL teachers, students’ achievement, Technological Pedagogical Content Knowledge, pedagogy, ICT

## Abstract

Educators themselves and their knowledge are the most significant elements in learners’ success and achievement; however, there is little information about the specialized knowledge held by educators. Recently, conceptualizing educator knowledge has become a complicated issue that involves that the comprehension of important basic phenomena, such as the procedure of educating and learning, the notion of knowledge, and the way educators’ knowledge, has become practical within the class. Technological Pedagogical Content Knowledge (TPACK) was presented as a theoretical structure for the knowledge base because educators have to efficiently instruct with technology and it is regarded as a valuable framework for explaining and understanding technology integration into various academic environments, such as English as a Foreign Language (EFL) class. This paper is intended to review the related literature on the role of an English teacher using the TPACK framework for teaching English and their role in students’ achievement. Based on the related literature, there is a significant and direct association between EFL teachers’ TPACK and EFL students’ achievement. Succinctly, some recommendations have been proposed for educational stakeholders.

## Introduction

Academic success takes place when learners learn and continue building talents, knowledge, and passion for learning within their life (Bransford et al., 2000). Educators are the most significant school-based elements due to their responsibilities regarding learner achievement (Keller et al., 2017) and studies confirm that educators and quality of education are the most significant indices of learners’ success. The longer learners work with successful educators, the greater will be their deliberate fulfillment (Guerriero, 2014). Learners’ success generally relies on the educator’s capability to teach each learner, cooperate individually, and begin building and assembling their own capacities, capabilities, and knowledge (Ali et al., 2020). English teaching in its recent tradition has been considerably altered with the significant introduction of technology, which is the most significant motivation for each social and linguistic alteration (Shyamlee and Phil, 2012). Indeed, education is a difficult procedure where numerous complicated procedures are included, such as content selection, and class activity, thus, numerous educators hold that technology merging in the class aids them to conduct the learning procedure efficiently and solving the complicatedness of the education and learning procedure (Chamorro and Rey, 2013). Nowadays, school educators are coping with a modern era of learners who are growing along with modern technologies as universal instruments. The modifications are taking place quicker than could have been thought 20 or 30 years ago (Spector, 2010). Intelligent portable instruments and social networks are of interest to students within the present trend of learning, providing new facilities (Ooi et al., 2018). Nowadays, technology and education are not separately taken into account, and it is essential to integrate technology into education (Liao, 2007; Arifani et al., 2020). In addition, higher education institutes continue to extend their online provisions to satisfy learners’ demands and, in this regard, make universities available to more learners (Kampov-Polevoi, 2010). Consequently, educators and course developers are currently motivated to change more in-person courses to online platforms. Various internet-based technologies are highly well-known among learners (Manca and Ranieri, 2013), making a number of them addicted to using cell phones or other portable cellphone devices (Singh et al., 2017).

In schools, principals, educators, learners, and parents are significant beneficiaries of technology merging, and they require knowing and internalizing the reasons why technology merging is being conducted. Among such beneficiaries, educators greatly affect learners’ achievement (Wright and Akgunduz, 2018). Since educational technology lacks a self-renewal characteristic, educators need to educate the curriculum within the class, assess learners’ learning procedures, and merge technology into their instructions to enhance learners’ learning (Kumar et al., 2008). However, improving access to technology does not appear to be sufficient to cause an efficient usage of technology in teaching (Tondeur et al., 2016). Undoubtedly, studies in various fields pointed to educators’ pedagogical content knowledge (PCK) as a crucial component contributing to learners’ learning and achievement (Gess-Newsome, 2015). An increasing body of studies indicates that learners’ achievement is significantly affected by the educator’s quality regarding content knowledge (CK) than by learners’ previous educational record or a school where a learner goes to (Ishola and Udofia, 2017). Educational achievement also alludes to the knowledge that learners obtain and the competencies that they have gained in educational centers (Stofile, 2017).

Teachers and parents mostly try to employ technology in the educational space as an essential component of getting learners ready for the constantly altering demands of the workforce in the future (Farrell and Hamed, 2017). Currently, most educators are not as overwhelmed by technology as their learners, and studies indicate their absence of knowledge to completely integrate technology in their class (Koehler et al., 2014). It can be stated that education is a multidimensional human effort that includes a complicated, moment-by-moment interaction of various classes of knowledge. Educators’ knowledge, pedagogical skill, and reasoning are important in enhancing learners’ learning achievement (Jacob et al., 2020). Efforts of incorporating technology into education have been mostly impeded by the absence of academic frames and models required for understanding such integration procedures (Mishra and Koehler, 2006). Embedding such technologies within the class needs educators to get technological knowledge, material, pedagogy, and how such main elements of education overlap (Archambault and Crippen, 2009). Moreover, the absence of educator knowledge, capabilities, skills, or experiences relevant to the utilization of technology in the teaching cycle has been determined as the primary impediment to technology incorporation (Oncu et al., 2008; Bingimlas, 2009; Chen et al., 2009). The technology incorporation impediments involve the absence of particular technological knowledge and abilities and the absence of technology-sponsored pedagogical and technology-related class administration knowledge and abilities. Thus, the methods relevant to technology incorporation in academia have transitioned from techno-oriented incorporation to techno-pedagogical one (Hew and Brush, 2007).

Educators’ technology integration in academic settings has been highlighted for years (Derakhshan et al., 2021). Numerous theories and models were suggested for enhancing educators’ knowledge and capabilities for technology integration. Consequently, this techno-oriented incorporation method emphasizes technology and has the objective of assisting educators with attaining the abilities and knowledge required to utilize different technologies. However, the techno-pedagogical incorporation method is contingent on pedagogy and sets both pedagogy and technology in practice in the incorporation cycle. One of the techno-pedagogical incorporation methods in the domain of technology incorporation in academia is the Technological Pedagogical Content Knowledge (TPACK) system that represents the knowledge needed to employ technology in an academic environment so that they are valid in terms of context and proper in terms of pedagogy (Abbitt, 2011). TPACK is primarily described as an educator knowledge framework for technology integration. Educator knowledge is described as a multifaceted relation and connection among three factors of knowledge in the TPACK agenda: material, education, and technology (Koehler and Mishra, 2009). Adding technological tools to education is not enough by itself for technology merging; a (TPACK) structure is crucial to embed technology, pedagogy, and material knowledge into the model (Koehler and Mishra, 2009).

Even though TPACK is a novel concept, it is a notion examined before by various scholars as a notion. It can be said that the research carried out by Keating and Evans (2001) was one of the initial ones in which the notion of TPACK was utilized. The term, Pedagogical Technology Knowledge (PTK), has a definition parallel to TPACK (Guerrero, 2005). PTK was characterized as technology-relevant teaching knowledge and highlighted as a novel area of expertise in the institutional schemes of educator knowledge. Furthermore, these notions are documented in the relevant literature as “Pedagogical Content Knowledge of Technology” (Margerum-Leys and Marx, 2002) and “techno-pedagogical skills in pre-service teachers” (Beaudin and Hadden, 2004). Accordingly, TPACK is implemented which makes an effort to make the boundaries of this knowledge, considering all three parts of knowledge and giving the teachers along with academic provide procedures to begin and assess the efficiency of technology incorporation in the procedure of teaching English. It is observed that using proper technology by educators positively affects learners’ achievement. For instance, Oztan (2012) found that using intelligent boards enhanced the success of 7th-grade learners in the “work, power, and springs” field in science and technology that can affect their achievement. In line with this, Tatar et al. (2013) conducted a study and found that the computer-assisted education by the educator positively affected the learners’ educational achievement. Another study carried out by Bugueño (2013) proved that using TPACK encouraged active language teaching in an English as a Second Language (ESL)/English as a Foreign Language (EFL) classroom.

As technology merging is a significant section of education reforms, TPACK has been flourishing in the study field. Several research and reviews have been done in the arena of academic technology, specifically displaying subjects and procedures in the TPACK field in the particular time covered (Chai et al., 2013; Voogt et al., 2013; Willermark, 2018). However, along the lines of the researcher’s knowledge, the role of TPACK on EFL students’ achievement has not been examined in the literature and most cases are about teachers.

## Review of the Literature

### Technology Integration

Technology integration is defined as employing technology as an instrument for improving learners’ learning, higher-level comprehension of course material, and developing high-degree thinking competencies (Spazak, 2013). Technology integration in education is described as integrating the process of learning and educating with proper technology for the goals, comprising of assessing lessons and learning results (Wachira and Keengwe, 2011). Nowadays, technology is immensely integrated into the daily life of learners and it needs to be completely taken into account in academic activity. Learners are overwhelmed by using personal computers (PCs), mobiles, applications, video games, music, and social media. Such technology integration in the learners’ lives should not be limited to the classroom. They are willing to see such technologies embedded in the class, in part, since they have learned using such instruments and their lives are highly interconnected with such technologies (Prensky, 2007).

### Technological Pedagogical Content Knowledge

The TPACK model emphasizes a holistic perspective of merging the three factors, which include technology, education, and CK, in education and learning settings. The TPACK model determined the complicated associations between the three factors, which are mutually bound or interacting in merging the Information and Communications Technology (ICT) in education. These main three elements intersect each other to form three mixtures, which include sections of Technology, technological pedagogical knowledge (TPK), PCK, and Technological Content Knowledge (TCK) (Chai et al., 2013). Technology knowledge (TK) alludes to knowledge regarding the possibility of fundamental or standard technologies, such as chalkboards and textbooks and new advanced technologies that include PCs, the internet, and virtual video employed in education and learning settings. This knowledge also comprised the capability to do specific technologies in education and learning procedures (Mishra and Koehler, 2008). CK is related to the educational employe’s knowledge or comprehensiveness regarding the material that these educational employes will educate, such as Science, Mathematics, History, and the English language. CK pertains to the subject contents, specificity, or features and uses in various standards. Concerning such conditions, educational staff requires implementing or applying various methods or approaches in the processes of education and learning (Mishra and Koehler, 2008). Pedagogical knowledge (PK) addresses the tactics, approaches in education, and learning procedures to gain learners’ learning results and goals in education. In such factor of TPACK, PK also alludes to implement lesson scheduling, plan procedures, manage educational resources, and assess learners. This overall form of competencies included the knowledge of methods used in the class environment, knowledge of the identity of the goal learners, and the tactics or approaches for evaluating learners’ comprehension of the subject. The PK permitted the educational staff to figure out and evaluate how learners obtain and build their knowledge in education and learning settings (Chai et al., 2013).

Of all such things, the TPACK element is the framework foundation since it is seen within the intersection of all the elements. TPACK element is described as a mixed knowledge that an educator should possess regarding the usage of educational and technological knowledge together in the education of a specific material for academic technology integration (Schmidt et al., 2009). The element of TPACK is described as a PCK transformation comprising field-specific tasks and topic-specific tasks. That is, the TPACK element alludes to “an educator’s” knowledge of how to organize and mix the usage of subject-specific tasks and topic-specific tasks using the modern technologies to ease learners’ learning (Cox and Graham, 2009). In their study, So and Kim (2009) defined the TPACK element as “recognizing how to provide subject matter with technology in pedagogically proper methods.”

Technological Pedagogical Content Knowledge theory was designed to state the series of knowledge that educators require to instruct their learners efficiently and to employ technology (McGraw-Hill, 2019). It tries to determine the role and function of knowledge needed by educators for technology integration in their education while coping with the complicated, multidimensional nature of educator knowledge (Valtonen et al., 2020). Educators’ education and information emerge from the setting of class teaching, which is context-specific, and they are spread across people, others, and instruments (Putnam and Borko, 2000). By associating with students, material, and technology instruments, educators learn from experience since they carry out their own teaching practices at school. Among these surrounding elements, researchers have mentioned that educators’ convictions have a crucial function in TPACK knowledge growth (Ertmer and Ottenbreit-Leftwich, 2010).

### Students’ Achievement

Achievement indicates particular learning achievement, which alludes to those knowledge, competencies, and comprehension, which originate from a specific school course (Ugwu, 2011). Later, it is shown that such learning is not easily obtained without a particular school or school experiences with a specific topic. Achievement examinations are developed to assess the result of the degree in a particular area or profession that a learner had done recently (Ugwu, 2011). Educational achievement is specified by the learners’ exam achievement points. Consistent with Ali (2013), educational achievement is a measure of the level of accomplishment in doing particular activities in a topic or area of study by learners after an experience of learning. It is the result of education that shows how well a learner or class of learners is/are academically performing. Learners’ educational achievement is described as the degree to which learners gain their short- or long-term educational purposes (Nja et al., 2020). Theoretical achievement is described as superiority in all educational fields, class, and complementary tasks (Kpolovie et al., 2014). Educational achievement refers to learners’ capability to successfully gain short- or long-term objectives (Rivkin et al., 2005).

## Conclusion

Teaching is a dynamic procedure and sometimes requires proper alterations in the education system. In a technology-led setting, educators require equipment with the competencies needed to be similar to their contemporaries worldwide. In both, the developed and developing nations, the governments are investing in offering computer infrastructure within schools. Either the state or central governments in different countries make remarkable investments in infrastructure development in schools for education through technology. A large number of software corporations are also helping toward training the educators in computer competencies and preparing customized courses for learners in different fields. What matters more is that, based on the research, technical competencies alone are insufficient for efficient education with computers, to make education constructive, it is essential to redesign the education tactics. When educators merge technology into education, their learners get more engrossed in the field (Schrum et al., 2007). In line with the review of literature, educators with higher knowledge in academic computer usage have higher prospects for learner’s learning, and using computers and academic technologies can assist in the enhancement of learners’ functions.

The literature review shows that TPACK-informed learning classes and apps were understood to be contributing and efficient as Chai et al. (2013) specified the TPACK frame as a platform in developing technology-improved scholastic settings. Contrary to the previous traditional classes, the professional development program highlights EFL educators’ PCK (Cabell and Hwang, 2020; Yazdanmehr et al., 2020; Metscher et al., 2021), but since the professional development program has moved from the conventional in-person to online-based apps, current professional development is concentrating on educators’ TPACK (Sahin, 2011; Tafazoli et al., 2019).

In summary, it can be stated that TPACK is fundamental to adequate education with technology, which needs comprehending the representation of notions employing technologies; pedagogical methods that employ technologies in practical methods to educate material; knowledge of what builds notions challenging or not difficult to study and how technology could benefit to reform a number of the problems that learners encounter; knowledge of learners’ previous theories and knowledge of epistemology; and knowledge of how technologies are employed to construct the current knowledge and to grow new epistemologies or reinforce the old ones. Therefore, it can be concluded that technology is regarded as an instrument that helps the learning procedure, meaning that representations of the topic can be improved through improving technology. In addition, TPACK is a constructivist method since the scholars recommended that technology is employed to support learning. This is suggested since technology can be a method to aid learners comprehend challenging notions relying on how it is merged and employed within the class. Educators turn into facilitators who can present content based on the learners’ level and particular requirements through implementing the TPACK framework, when this framework is used in a language class that leads to learners’ achievement. Furthermore, the TPACK model empowers educators to move the content knowledge to the learners and supports learners to learn better through practicing and experiencing how to cope with the technological concept (Misirli, 2016). Indeed, merging technology in the class, particularly for the English language class, has a worthy effect on the learning procedure and also on the students’ achievement (Lubis, 2018).

## Implications and Suggestions for Further Research

Learners can learn through various tasks within and outside the class by using virtual apps to learn the material and complete their educational tasks. Educators can also improve their pedagogical competencies by using virtual apps and making their education efficient. An educator can design various tasks to integrate technology with material and pedagogy to build an efficient learning setting for learners, which can assist their function better. Instead of assisting educators to merge technology into education, educators need to have the relevant knowledge to know how to employ technology to transform education and provide chances for learners’ achievement.

All educators of all competencies have to embed TPACK in their activities because it can focus on increasingly fascinating education by technology and provide educators with a system to think about what viewpoints they may need support with. The progress of educators’ TPACK needs to be through an embedded method where technology, education approach, and content knowledge are coped with together rather than separately. English teachers who cannot assimilate technology into their job experiences will be old-fashioned (Bugueño, 2013). Therefore, educator trainers have to attempt to present professional development for teachers through educational programs that look for increasing the usage of technology or providing further online classes, starting with concentrating on technology competency education, namely, how to upload a PowerPoint online, convert speeches into podcasts, or placing multiple-choice tests into a bank of the test. Educator trainers need to continue to offer chances for educators to continue to construct and improve their TPACK with different technologies, various material fields, and various educations.

When planning for education, educator trainers can take into account TPACK for designing courses and professional development chances. Technology can strongly interplay with material and educator training programs need to make educators ready to establish educational connections between those technology affordances and English teaching. It is recommended that educators integrate technologies in different manners to encourage learners to embed technologies in their learning procedure for the better comprehension of any subject, materials, and other assignments that can be performed through TPACK for a better result of learners’ success. The review of the related indicates that it is recommended that pre-service educators need exposure to technological instruments (that is simulations, digital laboratories, and virtual academic games) in educator training programs to grow their TPACK and to merge technology in their future paths (Canbazoglu Bilici et al., 2016). The teachers need to get themselves engaged in several professional learning chances to concentrate on developing online courses and the focus of their courses was basically on the technology: web-based development, library assistance, and PowerPoint podcasts. Educators with TPACK could prepare virtual presentations within the class setting based on the level at that learners are taught in the field. Pre-service educators have to acquire higher experience in employing technology, which can be gained by presenting more courses that encompass tasks and projects according to embedding technology’s usage in English education. Voogt et al. (2013) believe that educators need to be acquainted with different pedagogical methods and proper methods to employ ICT to help in developing their learners’ 21st-century competencies. Nowadays, extending learning chances through technology is a vital competency for English educators.

Individuals usually tend to do things in the manner they learned them. This has implications for educator’s education as well. Since using virtual technology in education is an innovation, the current educator trainers would not have employed it during their training. Therefore, they have two responsibilities of updating themselves about the methods of employing virtual equipment optimally for educating learning procedures along with conveying the information and executing it in the pre-service teaching. This review is central because the TPACK structure informs the beneficiaries regarding the development of technology-improved classes and apps. For instance, course educators can get conscious of how to merge the forthcoming technology into the syllabus; computer designers can get a better realization of how to construct efficient cellphone language-learning platforms; and academic institutes and agencies may get aware of how to suggest distance-learning apps proper for educators, specifically in the event of struggling with COVID-19 disease. These TPACK-improved programs can save educators’ time and attempt in fostering the associations between technology, education, and material.

Educator training programs need to offer technology laboratories that are accessible for pre-service educators so that they could turn into higher-skilled individuals in technology with more experience. Many educator trainers should apply the TPACK structure to develop syllabuses and expert training workshops for enhancing pre-service and in-service educators’ technology merging activity. Indeed, through workshops, conferences, and different tasks, pre-service educators can learn about employing several new technological instruments and contents during the learning procedure and adjust them to the present program.

Educational staff is needed to be provided with the technological competencies and to employ these technologies efficiently in the development of education (Chai et al., 2013). Examples of competencies needed are knowledge of running the software and hardware in the computer, ability to use sets of productivity software instruments, such as spreadsheet programs, browser of Internet, word processor, communicating utilities, and presenting slides. In addition, TK was also engaged with knowledge on installing parts of computer software and hardware.

Further studies are suggested to assess the way educator preparation programs make new educators ready with technology and improve learning in the whole learners. Comprehending the relationship between an educator’s TPACK and their learners’ achievement can support the provision of new educators and determine areas appropriate for future technology assets and professional development.

## Author Contributions

GD took responsibility for writing. LJ developed the theoretical framework. HC designed the research. All authors contributed to the article and approved the submitted version.

## Conflict of Interest

The authors declare that the research was conducted in the absence of any commercial or financial relationships that could be construed as a potential conflict of interest.

## Publisher’s Note

All claims expressed in this article are solely those of the authors and do not necessarily represent those of their affiliated organizations, or those of the publisher, the editors and the reviewers. Any product that may be evaluated in this article, or claim that may be made by its manufacturer, is not guaranteed or endorsed by the publisher.
